# Fiberoptic-Coupled Spectrofluorometer with Array Detection as a Process Analytical Chemistry Tool for Continuous Flow Monitoring of Fluoroquinolone Antibiotics

**DOI:** 10.1155/2020/2921417

**Published:** 2020-02-07

**Authors:** Nader Shokoufi, Maryam Vosough, Mona Rahimzadegan-Asl, Atefeh Abbasi-Ahd, Mahsa Khatibeghdami

**Affiliations:** ^1^Analytical Instrumentation and Spectroscopy Laboratory, Department of Green Technologies, Chemistry & Chemical Engineering Research Center of Iran, Tehran 14968-13151, Iran; ^2^Cancer Center, University of Illinois, Chicago, IL 60612, USA

## Abstract

Nowadays, there is an increasing need for sensitive real-time measurements of various analytes and monitoring of industrial products and environmental processes. Herein, we describe a fluorescence spectrometer in continuous flow mode in which the sample is fed to the flow cell using a peristaltic pump. The excitation beam is introduced to the sample chamber by an optical fiber. The fluorescence emitted upon excitation is collected at the right angle using another optical fiber and then transmitted to the fluorescence spectrometer which utilizes an array detector. The array detection, as a key factor in process analytical chemistry, made the fluorescence spectrometer suited for multiwavelength detection of the fluorescence spectrum of the analytes. After optimization of the experimental parameters, the system has been successfully employed for sensitive determination of four fluoroquinolone antibiotics such as ciprofloxacin, ofloxacin, levofloxacin, and moxifloxacin. The linear dynamic ranges of four fluoroquinolones were between 0.25 and 20 *μ*g·mL^−1^, and the detection limit of the method for ciprofloxacin, ofloxacin, levofloxacin, and moxifloxacin were 81, 36, 35, and 93 ng·mL^−1^, respectively. Finally, the proposed system is carried out for determination of fluoroquinolones in some pharmaceutical formulations.

## 1. Introduction

The Process Analytical Chemistry (PAC) is the application of analytical chemistry using specific techniques, algorithms, and sampling equipment which addresses the problems of chemical processes [1, 2]. In traditional analytical process, sampling was carried out manually, and then these samples were transferred to quality control laboratories [3]. Sometimes this approach was harmful, dangerous and also expensive, and ultimately did not result in good data in terms of accuracy. Also, in terms of time, it could not adequately demonstrate the process conditions. The delay in the analysis results prevented real-time and online analysis. Over time, it became clear that real-time measurements would provide time information in a process, so instead of offline measurements, continuous flow analytical devices were applied for online monitoring [4–8]. In continuous monitoring, direct connection of the sampling system, and the system itself, enables us to automate and control the collection of data and their analysis [5, 9]. The Process Analytical Technology (PAT) [10, 11] was defined in 2004 by the Food and Drug Administration (FDA) to support innovation in the industry quality control. PAT has many applications in the pharmaceutical and antibiotics manufacturing [12–25], chemical [26, 27], petrochemical [28], and food industries [29–33]. In addition, there are many recent progress in real-time monitoring of cultivations in bioreactors and cell culture process [34–41], fermentation [42, 43] and biological process [44], and electrochemical [45, 46] and protein purification [47]. Using PAT enables us to get a deeper understanding of the process. The process knowledge can increase the product integrity and production efficiency while it can reduce impurities and undesirable crops and costs which all lead to cost reduction eventually. By having additional information and knowledge about a process, it is possible to understand the parameters that influence the process and its quality control [15, 48].

Fluoroquinolones are among the most important antibacterial agents which were developed in the 80s and are now widely used in medicine and veterinary medicine [49]. Therefore, several analytical procedure has been reported for determination of fluoroquinolones including high-performance liquid chromatography (HPLC) [50–62], spectrophotometry [63], fluorimetry [64–73], flow-injection based on chemiluminescence [74–76], chemiluminescence [77, 78], terbium-sensitized luminescence [79], solid-phase spectrofluorimetry [80, 81], capillary electrophoresis [82, 83], convenient magnetic solid-phase extraction procedure coupled with capillary electrophoresis [84], solid-phase microextraction coupled with liquid chromatography-tandem mass spectrometry [85], liquid-liquid microextraction [86], polarography [87], and colorimetry [88] But, there is no report for application of PAT  or PAC for online monitoring of fluoroquinolones.

Hence, the main objective of this study is to develop a method for chemical analysis and monitoring of four fluoroquinolones (ciprofloxacin, ofloxacin, levofloxacin, and moxifloxacin) at continuous flow mode using a fiberoptic fluorescence array detecting system in pharmaceutical products.

## 2. Materials and Methods

### 2.1. Chemicals

Fluoranthene (a poly-aromatic hydrocarbon composition) with a purity of 98% was purchased from Sigma-Aldrich (St. Louis, MO). Spectroscopy-grade methanol was obtained from Merck (Darmstadt, Germany). Deionized water was produced by Millipore device (France). Active pharmaceutical ingredients (API) of the antibiotics ciprofloxacin, ofloxacin, levofloxacin, and moxifloxacin were commercially purchased from both Droupakhsh and Hakim Pharmaceutical Companies (Iran). The drug samples including intravenous infusion of 0.2% ciprofloxacin (Samen Mashhad Pharmaceuticals Company, Iran), ocular drop of 0.3% ciprofloxacin (Ciplex from Sina Darou Company, Iran), 200 mg ciprofloxacin tablet (Tehran Pharmaceuticals Company, Iran), ocular drop of 0.5% of levofloxacin (Oftaquix from Santen Pharmaceuticals Company in Finland), 500 mg tablet of levofloxacin (Tavanex) (Abidi Pharmaceutical Company, Iran) and 200 mg tablet of ofloxacin (Rouz-Darou Pharmaceutical Company, Iran) were also commercially purchased.

### 2.2. Apparatus

The florescence spectra were obtained by Array spectrophotometer model USB4000-FL with the light source of deuterium lamp model DH-2000 series and xenon model PX-2 (Ocean Optics Company, USA). The flow cell model FIA-SMA-FL-ULT series and the optical fibers model P600-2-SR were also from Ocean Optics (USA). The utilized optical fibers cover a wavelength region of 300^−1^100 nm and have a core diameter of 600 *μ*m. The flow adjusted by Peristaltic pump model BT100^−1^F (Longer Precision Pump, China).

### 2.3. General Procedure

The stock solution of 1000 *μ*g·mL^−1^ of four standard samples of antibiotics, including ciprofloxacin, ofloxacin, levofloxacin and moxifloxacin were separately prepared in 2 : 1 ratio of methanol/deionized water. Then, the dilution solutions of each were daily made and at continuous flow mode fed to the flow cell and detected by a fiber-optic fluorescence arrays system.

### 2.4. Fluorescence Array System in Continuous Flow Mode

**Figure 1:**
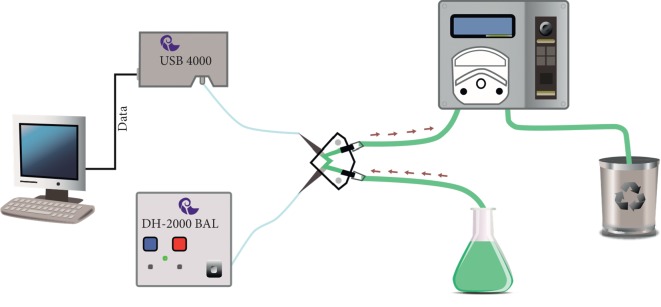
Experimental setup for continuous flow measurements using spectrofluorometric fiberoptic array.

In continuous flow monitoring, the flow of sample is continuously fed into a fluorescence flow cell by a peristaltic pump. Two fiberoptic probes were utilized to direct the light from light source to the flow cell and the fluorescence array from flow cell through the detector. Data are recorded and displayed on the computer software. The utilized laboratory set is presented in Figure 1.

### 2.5. Procedure for Pharmaceutical Samples

In order to validate the optimized method, the recovery value of different forms of commercial pharmaceutical samples of ciprofloxacin, ofloxacin, and levofloxacin were investigated. The pharmaceutical samples were included intravenous infusion of 0.2% ciprofloxacin, ocular drops of 3.8% ciprofloxacin, 200 mg ciprofloxacin tablet, 0.5% ocular droplet of levofloxacin, a 500 mg tablet of levofloxacin, and 200 mg ofloxacin tablet.

### 2.6. Preparation of Ciprofloxacin and Levofloxacin Solutions

For the analysis of ciprofloxacin, a stock solution of 2 *μ*g·mL^−1^ was prepared from 0.2% intravenous infusion of that. In order to evaluate accuracy of the method, other quantities of standard ciprofloxacin were added to the sample solution for recovery tests. All measurements repeated five times. The same procedure of sample preparation was repeated for measuring eye drops of ciprofloxacin 0.3% and levofloxacin 0.5%.

### 2.7. Preparation of Ciprofloxacin and Ofloxacin Solutions

For the analysis of ciprofloxacin samples, appropriate amount of ciprofloxacin tablets were weighed and transferred to a 100 mL container to form a stock solution of 1000 *μ*g·mL^−1^. Then, it was placed in an ultrasonic bath for 20 minutes. It was filtered with 0.22 micron membrane filters. Finally, it was diluted within the concentration range of the calibration curve and detected. Then, other quantities of standard ciprofloxacin were added to the sample solution for recovery tests. All measurements repeated five times.

The same procedure of sample preparation was repeated for measuring ofloxacin tablets.

## 3. Results and Discussion

### 3.1. Process Optimization

Optimization process was performed by optimizing flow velocity and integration time.

### 3.2. Effect of Flow Rate

**Figure 2:**
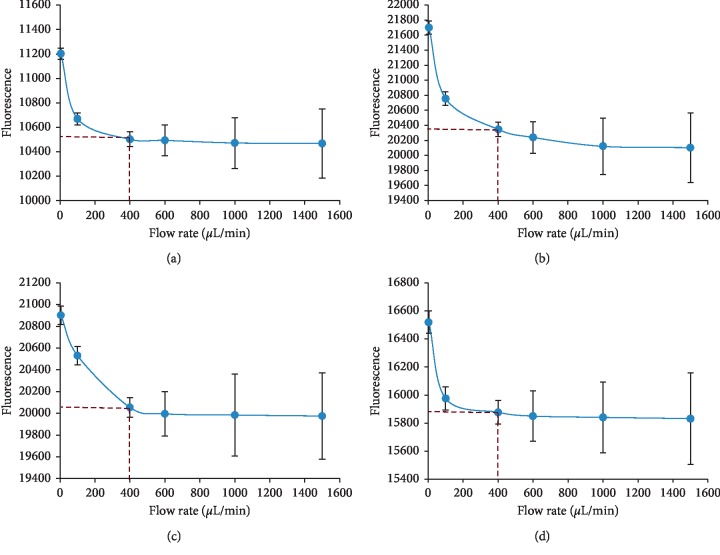
Effect of flow rate: (a) ciprofloxacin, (b) ofloxacin, (c) levofloxacin, and (d) moxifloxacin. Condition: concentration 5 *μ*g·mL^−1^; integration time: 600 mS.

The flow rates of the sample were optimized in the range of 5–1500 *μ*L·min^−1^ (Figure 2). The signal slightly decreased following the increase of the flow rate, it was considered that for the flow rate upper than 400 *μ*L·min^−1^, the obtained fluorescence spectra were unstable.

So, the flow rate of 400 *μ*L·min^−1^ was chosen for the best conformity with the signal stability and throughput of the sample.

### 3.3. Optimization of Integration Time

**Figure 3:**
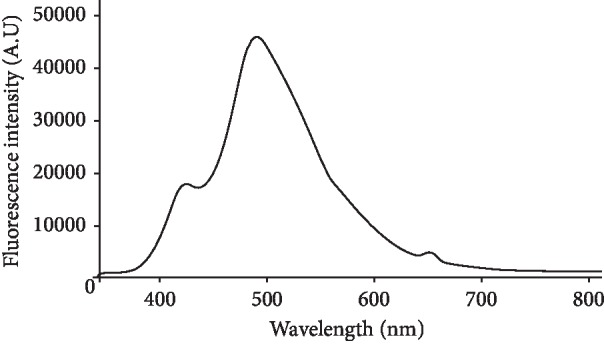
Optimization of integration time for levofloxacin. Condition: concentration 25 *μ*g·mL^−1^; integration time: 600 mS.

The integration time of the spectrometer (analogous to the shutter speed) was also optimized. The higher the integration time, the longer the detector monitors the incoming photons. For too low intensity, the value increased and vice versa. For the 25 mg·mL^−1^ of levofloxacin standard solution, with the integration time of 600 mS, the maximum intensity 45118.7 was achieved (Figure 3).

### 3.4. Characteristics of Emission Spectrum

**Figure 4:**
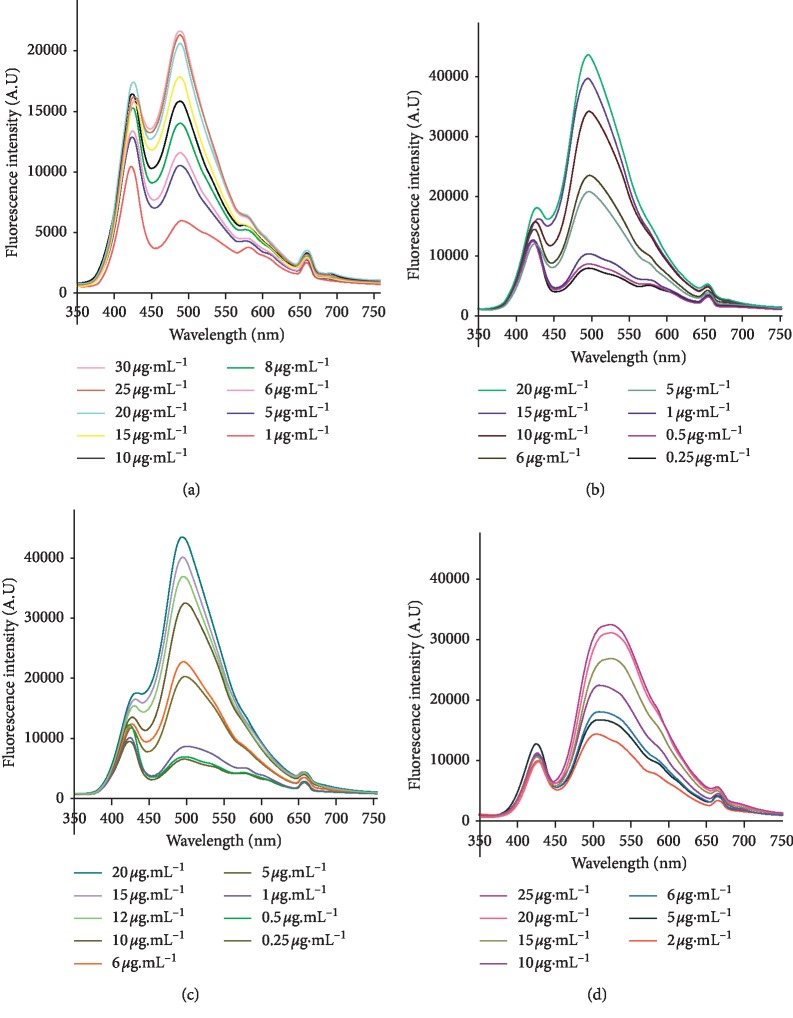
Fluorescence spectra of standard solutions: (a) ciprofloxacin, (b) ofloxacin, (c) levofloxacin, and (d) moxifloxacin. Condition: flow rate; 400 *μ*L·min^−1^; integration time: 600 mS.

The emission spectrums of various standard solutions of ciprofloxacin, ofloxacin, levofloxacin, moxifloxacin were recorded at the optimized conditions (Figure 4).

**Figure 5:**
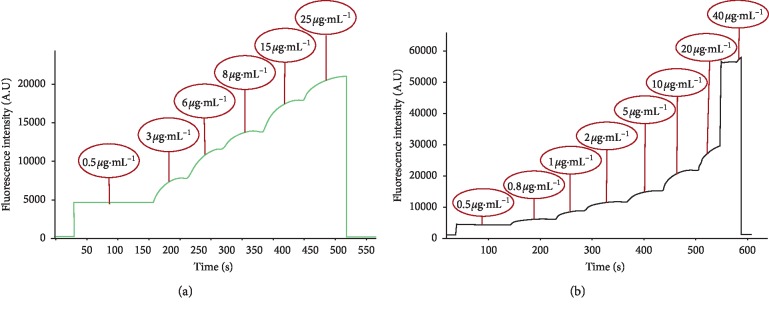
Fluorescence intensity vs. time: (a) ciprofloxacin, condition: flow rate: 400 *μ*L·min^−1^, integration time: 600 mS, *λ*_max_: 489 nm, and concentration range: 0.5–25 *μ*g·mL^−1^; (b) moxifloxacin, condition: flow rate: 400 *μ*L·min^−1^, integration time: 600 mS, *λ*_max_: 512 nm, and concentration range: 0.5–40 *μ*g·mL^−1^.

To demonstrate the capability of the method for continuous monitoring, 1 cc of standard solutions of ciprofloxacin and moxifloxacin from low concentration to high concentration were added to the flow cell, respectively, and changes of fluorescence intensity vs. time was monitored. As depicted in Figure 5, by increasing the concentration of ciprofloxacin and moxifloxacin the fluorescence intensity rises instantly.

### 3.5. Analytical Performances

**Figure 6:**
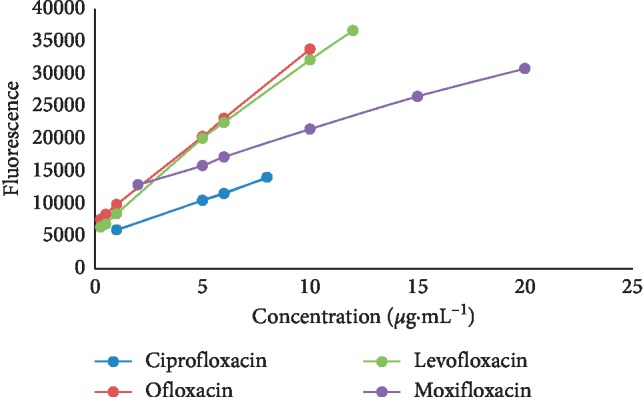
Calibration curve of standard fluoroquinolones. Condition: flow rate, 400 *μ*L·min^−1^; integration time: 600 mS.

Series concentrations of four antibiotics including ciprofloxacin, ofloxacin, levofloxacin, and moxifloxacin were used for drawing the calibration curves. A calibration graph of florescence intensity versus the sample concentration is presented in Figure 6.

The linear relationship, detection limit, and relative standard deviation of each are presented in Table 1.

In comparison with the other methods for fluoroquinolones determination, despite of simplicity of method, no labelling agent or additive for signal enhancement and monitoring of flowing sample, the proposed method had ng·mL^−1^ detection limit which is quite comparable with the most of other reported methods (Table 2).

### 3.6. Interference Study

To evaluate the selectivity of the developed method for the analysis of pharmaceutical preparations containing ciprofloxacin, the effects of some potential interference compounds (used as additive to pharmaceutical samples) such as fructose, glucose, sucrose, lactose, sorbitol, sodium citrate, magnesium stearate, talc, methyl cellulose, and starch on the efficiency of the presented method were studied. A standard sample solution of ciprofloxacin (5 *μ*g·mL^−1^) was analysed in the presence of the extra amount of coexisting substances. A compound was considered as noninterfering if the variation of its signal was less than ±5% in comparison with the signal in the absence of that.  Table 3 shows the results obtained. The results indicated that there were no significant interferences produced by these excipients substances on the proposed method for the determination of ciprofloxacin.

### 3.7. Measurements in Pharmaceutical Formulation

In order to evaluate the applicability of the optimized method for determination of fluoroquinolones, the recovery percent of ciprofloxacin, ofloxacin, and levofloxacin in pharmaceutical samples were investigated. The obtained results for commercial pharmaceutical samples are summarized in Table 3. The results show the potential of the developed method for the measurement of real samples (Table 4).

## 4. Conclusions

In this study, we have proposed a simple and sensitive method for quantitative measurement of fluoroquinolones medicines. The combination of flow system and array spectrofluorometric provides the ability to apply PAC for multiwavelength online monitoring of fluoroquinolones. The developed method was successfully utilized for analysis of ciprofloxacin, ofloxacin, levofloxacin, and moxifloxacin in low concentration range with detection limit of 35–93 ng·mL^−1^.

In addition to the fast detection time and automation, acceptable accuracy, and good reproducibility, the proposed method was used to measure fluoroquinolones in six forms of commercial pharmaceutical formulation as well and the obtained results showed the capability of method to be applied for online industrial analysis of real samples.

## Figures and Tables

**Table 1:** The analytical performance of the method.

Parameter	Moxifloxacin	Levofloxacin	Ofloxacin	Ciprofloxacin
*λ*_max_ (nm)	525	495	497	489
Linear range (*μ*g·mL^−1^)	2–20	0.25–12	0.25–10	1–8
Relative standard deviation^*∗*^ (*n* = 7%)	0.527	0.517	0.468	0.561
Detection limit (*μ*g·mL^−1^)	0.093	0.036	0.035	0.081
LOQ (*μ*g·mL^−1^)	0.308	0.1185	0.1155	0.269
Correlation coefficient	0.9979	0.9972	0.9999	0.9992

^*∗*^Concentration: 5 *μ*g·mL^−1^.

**Table 2:** Comparison of this method with other reported methods for determination of fluoroquinolones.

Method	Linear range (*μ*g·mL^−1^)	Detection limit (*μ*g·mL^−1^)
Spectrofluorometric [65]	0.02–2.2	0.006–0.016
HPLC-UV [89]	0.15 to 5	0.04–0.08
HPLC [57]	0.003–1.3	0.001
HPLC-fluorescence [90]	0.005–0.1	0.0005–0.005
Capillary electrophoresis [83]	5–20	1.1 and 2.4
Chemiluminescence [77]	1.98 × 10^−9^	0.003–7
HPLC–PDA [91]	0.1–10	0.03
Spectrophotometric [72]	0.5–25	0.084
HPLC [92]	0.0005–1	0.00007
This method	0.25–20	0.035–0.093

**Table 3:** Effect of some foreign interference compounds.

Tolerance limit [foreign substance]/[CIP]	Foreign substance added
80	Fructose, glucose, sucrose, lactose, sorbitol, sodium citrate, methyl cellulose
50	Magnesium stearate
40	Talc, starch

**Table 4:** Determination of ciprofloxacin, ofloxacin, and levofloxacin in pharmaceutical formulations.

Analytes	Tissue	Original (mg·mL^−1^)	Found (mg·mL^−1^)	RSD (*n* = 5%)	Content (*μ*g·mL^−1^)	Added (*μ*g·mL^−1^)	Found (*μ*g·mL^−1^)	Recovery (*n* = 5%)
CIP	Infusion	2	1.99 ± 0.003	1.91	0.04 ± 1.99	1	0.05 ± 3.01	102 ± 3
			1.92 ± 0.036	1.34	0.03 ± 1.92	3	0.19 ± 5.01	103 ± 2
			1.96 ± 0.016	1.06	1.96 ± 0.02	5	0.09 ± 6.97	100 ± 2

CIP	Eye drop	3	2.91 ± 0.028	8.80	0.17 ± 1.94	1	0.23 ± 3.02	108 ± 4
			2.93 ± 0.054	7.96	0.16 ± 1.95	3	0.14 ± 5.03	102 ± 3
			2.98 ± 0.006	5.68	0.11 ± 1.98	5	0.08 ± 7.01	100 ± 3

CIP	Tablet	2.5	2.47 ± 0.009	7.06	0.14 ± 1.98	1	0.23 ± 2.96	98 ± 4
			2.47 ± 0.010	4.34	0.09 ± 1.97	3	0.17 ± 5.10	104 ± 5
			2.41 ± 0.03	6.71	0.13 ± 1.93	5	0.21 ± 6.89	99 ± 4

OFLX	Tablet	2	1.98 ± 0.006	7.24	0.14 ± 1.98	1	0.15 ± 2.97	98 ± 4
			1.91 ± 0.043	7.63	0.15 ± 1.91	3	0.19 ± 5.06	104 ± 5
			1.95 ± 0.022	6.24	0.12 ± 1.95	5	0.19 ± 6.98	99 ± 4

Levo	Tablet	5	5.37 ± 0.075	5.81	0.13 ± 2.15	1	0.35 ± 3.17	102 ± 4
			5.32 ± 0.064	5.35	0.11 ± 2.12	3	0.29 ± 5.21	103 ± 4
			5.17 ± 0.034	7.39	0.15 ± 2.06	5	0.12 ± 7.17	102 ± 3

Levo	Eye drop	5	5.38 ± 0.077	5.01	0.11 ± 2.15	1	0.26 ± 3.13	98 ± 4
			5.18 ± 0.036	5.83	0.12 ± 2.07	3	0.16 ± 5.11	101 ± 4
			5.23 ± 0.046	7.54	0.16 ± 2.09	5	0.17 ± 7.08	100 ± 4

## Data Availability

The data used to support the findings of this study are available from the corresponding author upon request.
